# Initial breastfeeding attitudes and practices of women born in Turkey, Vietnam and Australia after giving birth in Australia

**DOI:** 10.1186/1746-4358-1-7

**Published:** 2006-04-07

**Authors:** Helen L McLachlan, Della A Forster

**Affiliations:** 1 Clinical School of Midwifery and Neonatal Nursing Studies, School of Nursing and Midwifery, La Trobe University, Bundoora, 3086, Australia; 2 Mother and Child Health Research, La Trobe University, 251 Faraday St, Carlton 3053, Australia

## Abstract

**Background:**

Cultural variations exist in the proportion of women who breastfeed. For some cultural groups, migration to a new country is associated with a reduction in the initiation and duration of breastfeeding. This paper describes the initial breastfeeding attitudes and practices of women born in Vietnam, Turkey and Australia who gave birth in Australia.

**Methods:**

The study included 300 women: 100 hundred Turkish-born, 100 Vietnamese-born and 100 Australian-born women who had given birth in a large public, tertiary referral maternity hospital between January 1998 and May 1999 in Melbourne, Australia. Women were interviewed in hospital, between 24 hours after the birth and discharge from hospital. Data were collected using a structured questionnaire with some open-ended questions. Only women who had a normal vaginal birth and who gave birth to a healthy baby were included in the study.

**Results:**

Almost all Turkish women initiated breastfeeding (98%) compared with 84% of Australian women. Vietnamese women had the lowest rate of breastfeeding initiation (75%), perceived their partners to be more negative about breastfeeding and did not value the health benefits of colostrum to the same extent as the other two groups. Forty percent of Vietnamese women gave their baby formula in hospital. The results of this study add to the previously reported finding that immigrant Vietnamese women have low breastfeeding rates compared with other groups.

**Conclusion:**

Despite the Baby Friendly status of the hospital where the study was conducted, major differences were found in breastfeeding initiation. Future research should develop and test interventions aimed at increasing breastfeeding initiation in Vietnamese women where initiation is low.

## Background

The World Health Organization (WHO) recommends that all infants should be exclusively breastfed from birth to six months of age followed by continued breastfeeding and the gradual introduction of solids [1]. In Australia, health policy makers support these recommendations [2]. Although breastfeeding initiation in Australia is around 83% [3,4] there are marked variations between subgroups. Australian studies have found, similar to others, that women are more likely to breastfeed if they are older [5], have more education [6], have higher income [4,7], and if they have social support [5,8]. Variations in breastfeeding rates among women who have migrated to Australia have also been reported [9-11].

Approximately 17% of all women giving birth in Victoria, Australia, are born in non-English speaking countries and an unknown proportion of Australian women, possibly an equivalent number, are of non-English speaking background [12]. Women born in Asia and the Middle East contributed to 9.3% and 2.3% respectively of all births in Victoria in 2002. This was an overall increase in Asian and Middle Eastern women from 7% in 1986 to 12% in 2002 [12].

Traditionally in Turkey almost all women breastfeed [13,14], however one study conducted in Istanbul reported that although 97% of women initiated breastfeeding, only 47% of women were exclusively breastfeeding at one week [13]. Some studies have reported a decreasing duration of breastfeeding in Turkey particularly related to maternal employment [15] and formula supplementation [14]. Less is known about breastfeeding initiation and attitudes of Turkish women following migration to a new country. One Australian study reported high initiation rates (97%) but two thirds of these women had stopped breastfeeding by two months [16].

In Vietnam, most women breastfeed and the majority breastfeed for at least one year [17,18], however a number of studies have reported that women born in Vietnam who have migrated to a new country have lower breastfeeding rates [9,10,19-25]. A number of these studies had small sample sizes [20,21,23] and most did not compare breastfeeding rates with other cultural groups. Two recent studies of Vietnamese women who had migrated to Australia reported higher initiation than the previous studies, (between 79% and 89%). In one of the studies half of the women had stopped breastfeeding by three months [26] but in the other 75% were continuing to feed some breast milk at this time [10].

Despite the unequivocal benefits of colostrum, the practice of withholding it from the newborn is widespread and variation exists between countries regarding length of time and reasons for withholding colostrum [27]. We have not identified any studies that reported Australian or Turkish women *not *feeding colostrum to their babies, however in Vietnam colostrum is reportedly often discarded [21,22].

A study was undertaken to compare the childbirth experiences of women born in Turkey, Vietnam and Australia on giving birth in Australia [28]. The aim of this paper is to describe and compare the early breastfeeding practices of Australian, Turkish and Vietnamese women giving birth in Australia, and to explore cultural differences in attitudes to breastfeeding.

## Methods

A comparative, descriptive research design was used.

### Participants

Three hundred women were included in the study: 100 Turkish-born, 100 Vietnamese-born and 100 Australian-born. To be eligible, Australian women had to have an English speaking background, defined as at least one parent born in Australia. The sample size of 300 was based on one of the major outcomes of the larger study, (experience of labour and birth) which was powered to detect a difference in satisfaction from 70 to 90% (80% power, 95% confidence interval) or from 70 to 50%. However the study was adequately powered to detect a difference from 60 to 80% in exclusive breastfeeding (91 women per group) or from 70 to 50% (103 women per group) (80% power, 95% confidence interval).

The three groups of women were selected for the study because of differences in cultural background, and because women from Turkey and Vietnam are large minorities in the population of women giving birth at The Royal Women's Hospital (RWH) in Melbourne, where the study was conducted. The hospital is a tertiary hospital with approximately 5000 births per year. Only women who had a normal vaginal birth without any major medical problems (such as severe pre-eclampsia/eclampsia, severe postpartum haemhorrhage, severe psychiatric disturbance, pulmonary embolus), who had given birth to a healthy baby (not admitted to neonatal nursery) and were booked as public patients, were eligible for inclusion into the study.

For each Turkish woman recruited to the study, the following Australian woman who gave birth and who met the eligibility criteria was approached in order to be sure that Australian women were recruited at a similar rate and over the same time period as the other women in the study. We aimed to recruit similar numbers of primiparas and multiparas to the study. During the initial phase of recruitment however, more Vietnamese and Turkish multiparas than primiparas gave birth, leading to the possibility that primiparas would be underrepresented in our sample. Following this, for each primiparous woman recruited, the following multipara who gave birth and met the inclusion criteria was approached.

### Data collection

All Turkish, Vietnamese and Australian women who had given birth were identified by a confidential, computer-generated report which identified country of birth. All eligible women were approached by the first author, HMc (excluding public holidays and annual leave). If the woman did not speak English, hospital interpreters or sometimes relatives were used to explain the study, gain consent and arrange an interview time. Women were interviewed in hospital, between 24 hours after the birth and discharge from hospital which is on average three days for primiparas and two days for multiparas. The interviews were conducted in a quiet place on the postnatal ward of the hospital. Vietnamese and Turkish speaking women were interviewed by Vietnamese and Turkish bilingual research assistants respectively and HMc conducted interviews with Australian women and Vietnamese and Turkish women who were fluent in English. Obstetric data were collected from the medical records.

The questionnaire was first developed in English and then translated into Turkish and Vietnamese by professionally trained and qualified translators, accredited by the National Accreditation Authority for Translators and Interpreters. Questionnaires were then translated back into English by a different qualified translator who had no knowledge of the original version, known as 'blind back translation' [29,30]. Using this method, the original questionnaire could be compared to the back translated version. The back translation was reviewed by HMc and discussed with the three qualified Turkish and Vietnamese interpreters. Discussion centred on the accuracy, meaning, clarity and appropriateness of the questions. There was some discussion related to the translation regarding 'problems attaching the baby to the breast' which was translated incorrectly to 'my child always wants to suckle'. This was translated and back translated a second time and was discussed with three qualified interpreters with a final back translation 'The baby has difficulty grabbing hold of the nipple with its mouth'. Original translations were accurate on the whole with few modifications made to the final translation.

In addition to detailed questions on the birth experience (reported elsewhere [34]), sociodemographic data were collected and questions were asked regarding women's breastfeeding intentions, attitude towards colostrum, current method of feeding, timing of first feed and breastfeeding problems during the first days after birth. Women were also asked about their partners' attitude towards infant feeding. The definitions used in this paper include 'breast milk only', that is, breast milk is the only nutritional intake, either at the breast or expressed; 'any breast milk' that is, a combination of breast milk and formula or 'formula', that is, formula only.

The Victorian Perinatal Data Collection Unit (PDCU) provided data on all Turkish, Vietnamese and Australian women who gave birth in Victoria during the study period to assess the representativeness of the study sample.

Ethical approval for the study was obtained from the Royal Women's Hospital and La Trobe University.

### Analysis

The women in each of the two migrant groups were compared with the reference group of Australian women and not with each other. The Australian women were used as the reference group in the analysis because the study was about the experiences and practices of migrant women in a new country. Comparisons between the groups were conducted using the chi square statistic to test the independence between factors in frequency tables, and t-tests were used to compare means of continuous data. Logistic regression analysis was used to explore the association between country of birth and breastfeeding outcomes to adjust for the effect of other important variables. The univariate association between each of the independent variables and the breastfeeding outcomes at the time of interview (ie feeding 'breast milk only' and 'any breast milk') was tested and only those that had a p value of <0.2 were included in the regression model with each outcome variable. Factors explored at the univariate level were number of years since migration to Australia (Vietnamese and Turkish women only), ability to understand spoken English, secondary education, higher education, parity, age, episiotomy, smoking, induction of labour, pharmacological pain relief and country of birth. All statistical analysis was undertaken using SPSS for Windows [31] or STATA [32].

## Results

### Participants

During the recruitment period (January 1998-May 1999), 289 Turkish, 274 Vietnamese and 4281 Australian women gave birth as public patients at the RWH, and of these 110 Turkish and 102 Vietnamese women met the study inclusion criteria and were invited to participate. Ten Turkish, two Vietnamese and no Australian women declined participation in the study.

### Background characteristics

The Turkish and Vietnamese women in the study were of approximately the same average age as other women born in these countries who gave birth in Victoria during the recruitment period (Turkish women: 27.7 vs. 27.8 years; Vietnamese women: 29.9 vs. 29.3). The proportion of married women was also similar (Turkish women: 99% vs. 95%; Vietnamese women: 82% vs. 81%) [33]. Australian women in our sample were on average younger (27.9 vs. 29.1 years) and significantly less likely to be married (45% vs. 74%) compared with the population of Australian women who gave birth in Victoria during the study period [33].

Table 1 shows that compared with the Australian group, Turkish women were more likely to be Muslim, multiparous, married, less educated, on a lower family income, and less likely to smoke. Vietnamese women were more likely to be Buddhist, married, older and on a lower family income compared with Australian women. Greater variation was observed regarding Vietnamese women's educational background, with a larger proportion having completed secondary school than Australian women but also a larger proportion having only limited education. Vietnamese women were less likely to smoke prior to becoming pregnant. Vietnamese women migrated to Australia more often as a refugee or for family reunion compared with Turkish women who migrated for marriage or better quality of life. The two migrant groups also differed in religious background with the majority of Turkish women being Muslim, and Vietnamese women Buddhist. Turkish women were more likely than Vietnamese women to speak English. All women in the study had a normal birth at term. The groups were similar in obstetric outcomes including augmentation and length of labour; however Vietnamese women were less likely to be induced (12%) compared with Australian (27%) and Turkish women (25%), and were more likely to have an episiotomy (29%) compared with Australian and Turkish women (16% and 18% respectively). Vietnamese women also had less pharmacological and non-pharmacological pain relief [34].

**Table 1:** Background characteristics of study participants

Characteristics	**Australian-born women **(n = 100)^1^	**Turkish-born women **(n = 100)^1^	**Vietnamese-born women **(n = 100)^1^
	n (%)^2^	n (%)^2^	n (%)^2^
**Marital status**			
Married	45	99	82
Defacto	47	0	11
Single/divorced/separated	8	1	7
**Age**, mean (sd)	27.9 (6.17)	27.7 (5.11)	29.9 (6.09)
**Education**			
Secondary school to final year	38	32	54
Secondary school but not completed	62	36	26
Primary school only	0	31	18
Did not attend school	0	1	2
**Further studies**			
Degree/diploma	9	9	14
Apprenticeship/traineeship	23	6	25
None	68	85	61
**Total family income per year (AUD)**^3^	(n = 90)	(n = 63)	(n = 43)
$20,000 or less	25 (27.8)	27 (42.9)	18 (41.9)
$20,000 – $30,000	22 (24.4)	16 (25.4)	8 (19.6)
$30,000 – $40,000	13 (14.4)	7(11.1)	7 (16.3)
More than $40,000	30 (33.3)	13 (21.6)	10 (23.3)
**Language of interview English**	100	38	27
**Ability to understand spoken English**		(n = 92)	(n = 92)
Very well		25 (27.2)	9 (9.8)
Fairly well		14 (15.2)	18 (19.6)
Not very well		47 (51.0)	48 (52.2)
Does not speak English		6 (6.5)	17 (18.5)
**Religion**			(n = 98)
Christian	53	0	22 (22.4)
Muslim	2	98	1 (1)
Buddhist	0	0	60 (61.2)
No religion	43	2	14 (14.3)
Other	2	0	1 (1)
**Reason for migration**			
Refugee		3	35
Family reunion		9	40
Marriage		59	23
Economic/quality of life		29	2
**Years in Australia, **mean (sd)		12.5 (9.4)	9.0 (5.3)
**Smoked prior to pregnancy**	51	30	2
**Primiparas**	41	30	41

1: n= 100 in each group unless otherwise stated

2: Percentages only reported when n not equal to 100

3: AUD – Australian dollars

### Breastfeeding outcomes of Vietnamese women

Significantly fewer Vietnamese women (60%) were exclusively breastfeeding during the first days after birth compared with Australian women 81% (Table 2). There was no statistical difference in the number of women who fed any breast milk at the univariate level (Vietnamese 75%, Australian 84%), however the difference became statistically significant after adjusting for confounders (see multivariate analysis below) (Table 2). Forty percent of Vietnamese women had fed their baby formula in hospital compared with 19% of Australian women.

**Table 2:** Method of feeding in hospital

**Outcomes**	**Australian-** **born women ** (n = 100)	**Turkish-** **born women ** (n = 100)	**Vietnamese-** **born women ** (n = 100)	**Unadjusted Odds Ratio** **(95% CI)**	p*	**Adjusted Odds Ratio** **(95% CI)**	p*
**Breast milk only**							
Turkish c/w Australian	81	92	-	2.70 (1.05,7.14)	0.04	2.90 (1.2, 7.03)	0.02
Vietnamese c/w Australian	81	-	60	0.35 (0.18,0.70)	0.002	0.29 (0.15, 0.56)	<0.001
Secondary education completed	38	32	54	1.74 (0.97, 3.09)	0.06	2.71 (1.43, 5.14)	0.002
No/poor English	0	53	65	0.51 (0.29, 0.88)	0.02	ns	
Any breast milk ***							
Turkish c/w Australian	84	98	-	9.33 (2.08,85.25)	0.001#	10.12 (2.25,45.5)	0.003
Vietnamese c/w Australian	84	-	75	0.57 (0.27,1.21)	0.16	0.47 (0.23, 0.98)	0.04
Secondary education completed	38	32	54	1.20 (0.98, 4.10)	0.06	2.93 (1.38, 6.22)	0.005
**Formula only**							
Turkish c/w Australian	16	2	-	0.11 (0.01,0.48)	0.001#	n/a	
Vietnamese c/w Australian	16	-	25	1.75 (0.82,3.74)	0.16	n/a	

Adjusted for country of birth, secondary education, ability to understand spoken English

* Australian women reference group

*** Combination of breast milk and formula

# Exact confidence limits reported

ns: not significant in final model

n/a: not presented as is opposite to 'any' breast milk calculations

c/w: compared with

Compared with Australian women, the Vietnamese women were less likely to have planned to breastfeed (90% vs 79%) and did not value the health benefits of colostrum to the same extent (Table 3). There were no differences between Vietnamese and Australian women in their perception of their partners' infant feeding preference. Of those Australian and Vietnamese women who were breastfeeding, there were no differences in the proportion of women who reported having a 'great' or 'good deal' of trouble breastfeeding (14% and 8% respectively). However of those women reporting having difficulty feeding, the reasons given for the breastfeeding problems varied, with Australian women being more likely to cite difficulties with attachment (83%) compared with the Vietnamese women (31%). Vietnamese women more often described sore nipples as their main problem (63%) compared with Australian women (17%) (Table 3).

**Table 3:** Breastfeeding attitudes, intentions and problems: Vietnamese-born women compared with Australian-born women

**Breastfeeding attitudes, intentions and problems**	***Australian-born*** ***women *n (%)†**	***Vietnamese-born*** ***women *n (%)†**	**Odds Ratio (95% CI)**	p*
**Planned to breastfeed**	(n = 100)	(n = 100)		
	90	79	0.42 (0.17,1.00)	0.05
**Breastfeeding preferred by partner (women's perceptions)**	(n = 95) 61 (64.2)	(n = 85) 62 (73.0)	1.50 (0.76,2.98)	0.27
**If breastfeeding, attitude towards colostrum compared with milk produced later**	(n = 84)	(n = 75)		
More healthy for baby	46 (54.8)	19 (25.7)	0.28 (0.13,0.58)	<0.001
Equally healthy	20 (23.8)	48 (64.9)	5.69 (2.71,12.06)	<0.001
Less healthy	0	1 (1.3)	**	
Not sure	18 (21.4)	7 (9.3)	0.38 (0.13,1.04)	0.06
**Breastfed within 1 hour of birth**	(n = 93)	(n = 68)		
	65 (69.9)	38 (55.9)	0.55 (0.27,1.10)	0.10
**Problems breastfeeding**	(n = 84)	(n = 75)		
Great/good deal	12 (14.3)	6 (8.0)	0.52 (0.15,1.61)	0.32
Moderate	17 (20.2)	10 (13.3)	0.61 (0.24,1.53)	0.34
Very little/none	55 (65.5)	59 (78.7)	1.94 (0.90,4.22)	0.09
**Type of breastfeeding problems**	(n = 29)	(n = 16)		
Attachment	24 (82.8)	5 (31.3)	0.09 (0.02,0.48)	0.002
No/insufficient milk	1 (3.4)	3 (18.8)	**	
Baby unsettled	4 (13.8)	1 (6.3)	**	
Sore nipples	5 (17.2)	10 (62.5)	8.00 (1.65,42.48)	0.006
Other	2 (6.9)	2 (12.5)	**	

* Australian women reference group

** Numbers too small to undertake statistical test

†Percentages only reported when n not equal to 100

### Breastfeeding outcomes of Turkish women

Almost all Turkish women breastfed their baby (98%) compared with 84% of Australian women (Table 2). Women born in Turkey were more likely to perceive that their partners wanted them to breastfeed (83%) compared with Australian women (64%) (Table 4). The majority of Turkish women considered colostrum to be healthier for the baby than breast milk (60%), which was similar to the views of the Australian women (55%).

**Table 4:** Breastfeeding attitudes, intentions and problems: Turkish-born women compared with Australian-born women

**Breastfeeding attitudes, intentions and problems**	***Australian-born women ***n (%)†	***Turkish-born women ***n (%)†	**Odds Ratio (95% CI)**	p*
**Planned to breastfeed**	(n = 100)	(n = 100)	1.48 (0.49,4.52)	0.61
	90	93		
**Breastfeeding preferred by partner (women's perceptions)**	(n = 95) 61 (64.2)	(n = 99) 82 (82.8)	2.69 (1.31,5.56)	0.005
**If breastfeeding, attitude towards colostrum compared with milk produced later**	(n = 84)	(n = 98)		
More healthy for baby	46 (54.8)	59 (60.2)	1.25 (0.66,2.35)	0.55
Equally healthy	20 (23.8)	4 (4.0)	0.14 (0.03,44)	<0.001
Less healthy	0	3 (3.1)	**	
Not sure	18 (21.4)	32 (32.7)	1.78 (0.86,3.68)	0.12
**Breastfed within 1 hour of birth**	(n = 93)	(n = 98)		
	65 (69.9)	63 (64.3)	0.78 (0.40,1.48)	0.50
**Problems breastfeeding**	(n = 84)	(n = 98)		
Great/good deal	12 (14.3)	32 (32.7)	2.91 (1.31,6.55)	0.007
Moderate	17 (20.2)	19 (19.4)	0.95 (0.43,2.09)	0.96
Very little/none	55 (65.5)	47 (48.0)	0.49 (0.25,0.92)	0.03
**Type of breastfeeding problems**	(n = 29)	(n = 51)		
Attachment	24 (82.8)	26 (51.0)	0.22 (0.06,0.72)	0.009
No/insufficient milk	1 (3.4)	9 (17.6)	**	
Baby unsettled	4 (13.8)	2 (3.9)	**	
Sore nipples	5 (17.2)	39 (76.5)	15.6 (4.39,61.55)	<0.001
Other	2 (6.9)	2 (3.9)	**	

* Australian women reference group

** Numbers too small to undertake statistical test

†Percentages only reported when n not equal to 100

Of the Turkish women who were breastfeeding, they were more likely to say they were having a 'good' or 'great deal' of trouble breastfeeding (33%) than women from Australia (14%). The reasons given for the breastfeeding problems also varied, with Australian women being more likely to cite difficulties with attachment (83%) compared with Turkish women (51%). Turkish women having breastfeeding problems more often described sore nipples as their main problem (77%) compared with Australian women (17%) (Table 4).

### Multivariate analysis

Regression analysis was used to explore whether country of birth differences in breastfeeding in hospital remained after adjusting for other potentially confounding factors. Number of years since migration to Australia (Vietnamese and Turkish women only), higher education, parity, age, smoking status, induction of labour, pharmacological pain relief in labour and episiotomy were not significantly associated with either any or exclusive breastfeeding at the univariate level. Secondary education was negatively associated with *any *and *exclusive *breastfeeding and poor/no ability to understand spoken English was negatively associated with *exclusive *breastfeeding. These were entered in regression models with country of birth and each of the two breastfeeding outcomes (Table 2).

Turkish women remained significantly more likely to give *any *breast milk (adjusted OR 10.12; 95% CI 2.25,45.5) and to exclusively breastfeed (adjusted OR 2.90; 95% CI 1.2,7.03) in hospital than Australian women.

Vietnamese women remained significantly less likely than Australian women to exclusively breastfeed their babies in hospital (adjusted OR 0.29; 95% CI 0.15,0.56). After accounting for confounders, Vietnamese women were also significantly less likely to give any breast milk than Australian women (adjusted OR 0.47; 95% CI 0.23,0.98).

For all women, completing secondary school was associated with an increased likelihood of giving *any *breast milk (adjusted OR 2.93; 95% CI 1.38,6.22) and *exclusively *breastfeeding (adjusted OR 2.71; 95% CI 1.43,5.14).

## Discussion

Overall, Vietnamese women had the lowest breastfeeding initiation, which was consistent with their breastfeeding intentions and they did not value the health benefits of colostrum to the same extent as Australian women. Turkish women were more likely to commence breastfeeding and they were more likely to perceive that breastfeeding was preferred by their partners when compared with Australian women. Turkish women also reported more breastfeeding problems.

The variation in the proportion of women breastfeeding between cultural groups found in this study is consistent with many other studies. Australian women breastfed at a similar rate (84%) to that reported in the most recent Australian national data using the 2001 National Health Survey, which estimated that the proportion of children breastfed at discharge from hospital was 83% [4].

### Vietnamese women

The results of this study add to the previously reported finding that immigrant Vietnamese women are less likely to initiate exclusive breastfeeding following migration to a new country [9,10,19-25]. Reasons previously cited for low breastfeeding rates among immigrant women are convenience [19,25], perceptions of impaired quality of milk [19,35] economic reasons such as the need to return to work [22,25], a decrease in social support, [22,25], perceptions that more affluent families do not breastfeed their own babies [22] and a desire to conform to the perceived cultural norm of the new country [35]. In this study, reasons for breastfeeding outcomes were not explored in-depth and it is therefore difficult to draw conclusions. The beliefs of people from many Asian countries that colostrum is not as healthy for the baby does not seem to have been changed by the strong evidence of its benefits [27]. Traditionally in Vietnam, the newborn is fed rice water or other liquid while the colostrum is expressed and discarded and the baby is then put to the breast around day three [20]. Perhaps the negative view of colostrum is one reason why breastfeeding Vietnamese women were more likely than Turkish or Australian women to give supplementary formula whilst in hospital. It is also possible that the lower rates of breastfeeding were only temporary because of cultural beliefs regarding colostrum.

### Turkish women

Our finding that 98% of Turkish women breastfed in hospital is consistent with the finding by Yelland and colleagues that 97% of Turkish women who had migrated to Australia, initiated breastfeeding [9]. Almost all Turkish women in this study were Muslim; in the Quran, mothers are encouraged to breastfeed for two years [36]. Despite reports of women from some migrant groups initiating breastfeeding less often following migration to a new country, this does not seem to be the case with Turkish women in this study. Studies of breastfeeding women in Turkey however have reported that women are ceasing breastfeeding earlier due to little support for breastfeeding in the workplace, a lack of maternity leave [15] and formula supplementation in hospital [14]. We do not have data on the duration of breastfeeding so we do not know if these factors influenced the Turkish women in our study.

Women were asked whether or not they thought their partner wanted them to breastfeed. Women born in Turkey were more likely to state that their partner wanted them to breastfeed (83%) than Australian (64%) and Vietnamese women (73%). This is consistent with studies which have shown that social support from a partner, or the influence of family, friends or health professionals affect infant feeding methods and rates of breastfeeding [37-39]. However, it is possible that women's partners were also influenced about infant feeding methods in the same way as the women themselves and therefore shared the same views.

Of those women who commenced breastfeeding, Turkish women were significantly more likely to say that they had trouble breastfeeding. This may be related to Turkish women acknowledging any difficulties they had and attempting to resolve them. There is a cultural expectation that Turkish women can and do breastfeed. It is also possible that Turkish women felt more able to express their difficulties. Another possibility is related to the level of breastfeeding support whilst in hospital. Midwives may offer less help to Turkish women either due to language difficulties or because of a perception that Turkish women usually breastfeed successfully. Australian women may have received more help because they asked for it or because they were more likely to be offered help.

Australian women's breastfeeding problems were usually related to difficulties with attachment compared with Turkish women who more often reported sore nipples. Australian women may have been more aware that problems with attachment may lead to sore nipples. 'Attachment problems' is a relatively newly derived term for unsuccessful breastfeeding and it is a term used by midwives.

### Care in hospital

The majority of breastfeeding women in this study breastfed within one hour of the birth. This finding may be related to the fact that the hospital in which the study was conducted had been accredited as a Baby Friendly Hospital (BFHI), a joint World Health Organisation (WHO) and United Nations Children's Fund (UNICEF) global initiative which encourages the first breastfeed within one hour of the birth [40]. However despite the BFHI status of the hospital there were marked differences between the groups in infant feeding during the first days after the birth, with forty percent of Vietnamese women giving their baby formula. The hospital environment is a key area where care providers may be able to help make a difference to breastfeeding initiation, particularly for women of non-English speaking background who are less likely to attend childbirth education classes [41].

A question that arises from our findings is how to deal with traditional cultural practices that do not encourage optimal health outcomes. In this study, the lower rate of breastfeeding may have health implications for Vietnamese mothers and babies. How health professionals tackle this issue is complex. Some cultural awareness and knowledge of the evidence of findings in studies such as this one may be essential to allow early interventions to occur. However Vietnamese, Turkish and Australian women are not homogenous cultural groups and should not be stereotyped as such. They will be influenced by a range of social, personal and cultural circumstances which are not generalisable and care providers need be sensitive and accepting of these differences.

Some studies have suggested targeting breastfeeding education programs for migrant women in western countries [42-44]. In a culture-specific education program to promote breastfeeding among Vietnamese women in Sydney, Australia, Rossiter [42] reported significant effects on knowledge, attitudes and initiation of breastfeeding, although the study had some design problems including using a convenience sample, questionnaires not being translated back into English and the researcher and interpreters being used as support people for breastfeeding problems experienced by women in the study. The study by Yelland and colleagues [10] found higher rates of breastfeeding among Vietnamese women who had attended an antenatal model of care designed specifically for Vietnamese women. The model employs midwives, medical practitioners, a community development worker and interpreters and operates with a philosophy of continuity and an individualised approach to care [10]. This may be a model which could be further explored.

In this study a variety of strategies were used in an attempt to ensure validity and reliability. These included translation, followed by 'blind' back translation by qualified translators, consultation with bilingual interpreters and translators, piloting and re-piloting of questionnaires, and careful selection, training and support of bilingual interviewers. However, working with translated research instruments is complex, and despite extensive and careful translation and back translation we cannot completely rule out the possibility that words such as those used as response alternatives may have slightly different meanings in the three languages and cultures. Although we used a rigorous translation process it is possible that differences in the perception of words such as 'attachment' for example, related to subtle translation differences.

Our results suggest that the Turkish and Vietnamese women in this study were more representative of the general Victorian populations from these countries than the Australian sample. One explanation of these findings was the selection of only public patients into the study. During the recruitment period, 35% of Australian women who gave birth in Victoria were admitted for the birth as private patients compared with only 4% of Turkish and 8% of Vietnamese women [33]. It is likely that if private patients were included in the study the disparity found between Australian women and Vietnamese women would have been greater, given the higher rate of breastfeeding among women receiving private maternity care in Victoria [45].

## Conclusion

We found significant differences in breastfeeding initiation between Turkish, Vietnamese and Australian women in this study. The hospital environment is one place where care providers have the opportunity to influence breastfeeding behaviour. Despite the hospital where the study was conducted being accredited as Baby Friendly, 40% of Vietnamese women fed their baby formula in hospital. Future research should include well designed randomised controlled trials to test interventions specifically aimed at increasing breastfeeding initiation among Vietnamese women.

## Competing interests

The author(s) declare that they have no competing interests.

## Authors' contributions

HMc collected and analysed data and drafted and revised the manuscript; DF undertook data analyses and contributed to revision of the manuscript.
